# Simple and Label-Free Detection of Carboxylesterase and Its Inhibitors Using a Liquid Crystal Droplet Sensing Platform

**DOI:** 10.3390/mi13030490

**Published:** 2022-03-21

**Authors:** Duy-Khiem Nguyen, Chang-Hyun Jang

**Affiliations:** Department of Chemistry, Gachon University, Seongnam-daero 1342, Sujeong-gu, Seongnam-si 13120, Gyeonggi-do, Korea; khiem80@gachon.ac.kr

**Keywords:** carboxylesterase, liquid crystals (LC), myristoylcholine, LC droplet-based sensor, inhibitor

## Abstract

In this study, we developed a liquid crystal (LC) droplet-based sensing platform for the detection of carboxylesterase (CES) and its inhibitors. The LC droplet patterns in contact with myristoylcholine chloride (Myr) exhibited dark cross appearances, corresponding to homeotropic anchoring of the LCs at the aqueous/LC interface. However, in the presence of CES, Myr was hydrolyzed; therefore, the optical images of the LC patterns changed to bright fan-shaped textures, corresponding to a planar orientation of LCs at the interface. In contrast, the presence of CES inhibitors, such as benzil, inhibits the hydrolysis of Myr; as a result, the LC patterns exhibit dark cross textures. This principle led to the development of an LC droplet-based sensing method with a detection limit of 2.8 U/L and 10 μM, for CES detection and its inhibitor, respectively. The developed biosensor not only enables simple and label-free detection of CES but also shows high promise for the detection of CES inhibitors.

## 1. Introduction

Liquid crystals (LCs) are promising materials for biosensing owing to their unique physical properties such as long-range orientational order, optical anisotropy, and molecular sensitivity [1,2]. Thus, LCs have been widely used to develop novel sensing systems for monitoring biochemical events [3,4]. LC-based sensing systems can transform chemical/biological events into optical responses that can be observed under a polarized light microscope (POM) [4,5]. Moreover, these sensing systems can operate in ambient environments without the need for molecular labels or electrical power [5,6,7]. Recently, LC-based sensors for monitoring various biochemical events have been developed and reported [5,7,8,9,10]. Among these, LC droplet-based sensors have been widely used to investigate enzymatic activity because the large surface-area-to-volume ratio of the LC droplets can increase the sensitivity of the detection [3,11]. The LC droplets were formed by simply dropping the solution of LC dissolved in organic solvents onto the surfaces of glass slides. The LC patterns exhibited bright fan-shaped appearances when in contact with non-surfactant solutions (e.g., deionized water), whereas dark cross textures were observed after introducing surfactant solutions [11,12].

Carboxylesterases (CESs) are an important class of enzymes that catalyze the hydrolysis of carboxylic esters and play a crucial role in the detoxification and metabolism of many drugs [13,14,15]. Moreover, the level of CES can serve as an indicator of hepatocellular carcinoma [16]. Thus, the accurate measurement of CESs is very helpful for better understanding their biological functions as well as for the early diagnosis of hepatocellular carcinoma.

To date, several detection methods, including fluorescence [14,15,17], chromatography [18], and luminescence [19,20], have been developed for CES detection. However, these methods have limitations including the need for complex instrumentation, labeling of samples, and long operating times. Recently, a simple and sensitive sensing system for CES detection based on a surfactant-decorated LC/aqueous interface was reported [21]. However, some drawbacks need to be addressed. The copper grids used to prepare LC/aqueous interfaces are easily oxidized and deformed, which limits their reusability [11]. Moreover, when preparing LC thin films, excess LCs are removed from copper grid waste materials.

**Figure 1:**
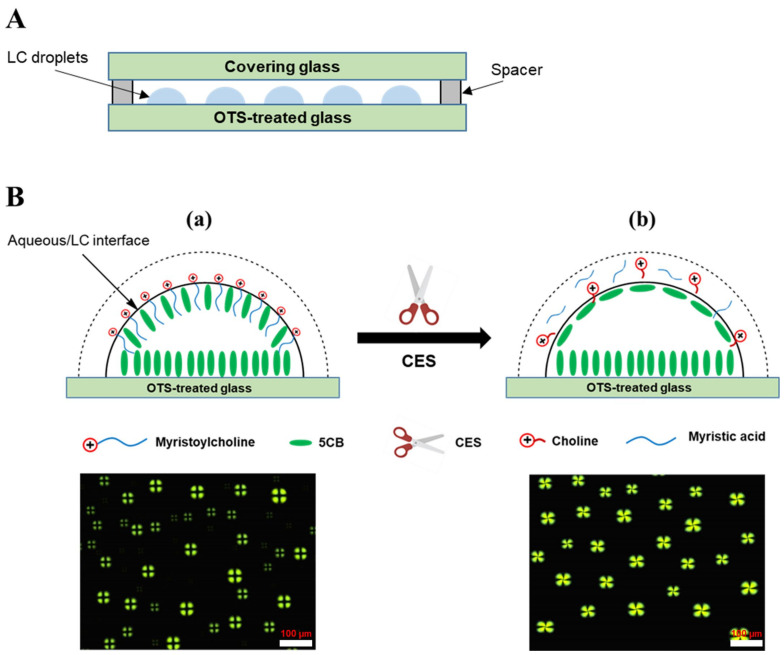
(**A**) Structure diagram of a liquid crystal (LC) cell; (**B**) Schematic illustration of the LC orientation transition at the aqueous/LC interface and the corresponding optical images: (**a**) after the addition of myristoylcholine chloride (Myr) solution; and (**b**) after introducing a mixture of Myr and carboxylesterase (CES).

In this study, we developed a simple and sensitive LC droplet-based biosensor for CES detection. The structure diagram and principle of the developed sensor are illustrated in Figure 1. The LC droplet patterns were formed by spreading 1.5 µL of a heptane solution containing 2% (*v*/*v*) 5CB onto the OTS-modified glass slides after the evaporation of heptane. OTS with a hydrophobic tail was used to align the 5CB molecules at the glass/LC interface in a homeotropic orientation. Myristoylcholine (Myr, the substrate of the CES enzyme) was used as a cationic surfactant to induce homeotropic anchoring of LCs at the LC/aqueous interfaces, corresponding to the dark cross appearances of the LC droplets observed under POM (Figure 1B(a)). However, in the presence of CES, the optical images of the LC droplet patterns changed to bright fan-shaped textures owing to the enzymatic hydrolysis of Myr by CES (Figure 1B(b)). Moreover, the effect of the inhibitor on CES activity was investigated. This biosensor enables simple and fast detection of CES and its inhibitors with high selectivity. 

## 2. Materials and Methods

### 2.1. Materials and Apparatus

The materials, reagents, and apparatus used in this study are listed in the Supplementary Materials section.

### 2.2. Preparation of Octyltrichlorosilane (OTS)-Modified Glass Slides

OTS-modified glass slides were prepared as previously described [22,23]. The details are provided in the Supplementary Materials section.

### 2.3. Preparation of LC Droplet Patterns and Fabrication of LC Cells

LC droplet patterns were prepared by dropping 1.5 µL of 5CB (2% (*v*/*v*) in heptane) onto the OTS-modified substrate. The solvent was evaporated at room temperature (~25 °C) and LC droplet patterns were formed. Subsequently, approximately 100 µL of aqueous solution was transferred onto the LC droplets at 25 °C. The LC cells were fabricated by pairing the covering glass slides and OTS-modified glass slides face to face (Figure 1A). Two small pieces of glass (26 × 0.5 mm, thickness = 1 mm) were used to produce a gap between two glass slides.

### 2.4. Preparation of the Aqueous Solution

All aqueous solutions were prepared in phosphate-buffered saline (PBS, 10 mM, pH = 7.4). During the CES detection assay, varying concentrations of CES were mixed with 10 µM Myr and incubated at 37 °C for 30 min. For the enzyme inhibition assay, a mixture of CES (0.1 µg/mL) and benzil at various concentrations was pre-incubated at 37 °C for 2 h. After the addition of 10 µM Myr, the mixtures were incubated at 37 °C for 30 min. Subsequently, 100 µL of the incubated solution was added to the LC droplet at 25 °C.

## 3. Results and Discussion

### 3.1. Optimization of the Myr Concentration

**Figure 2:**
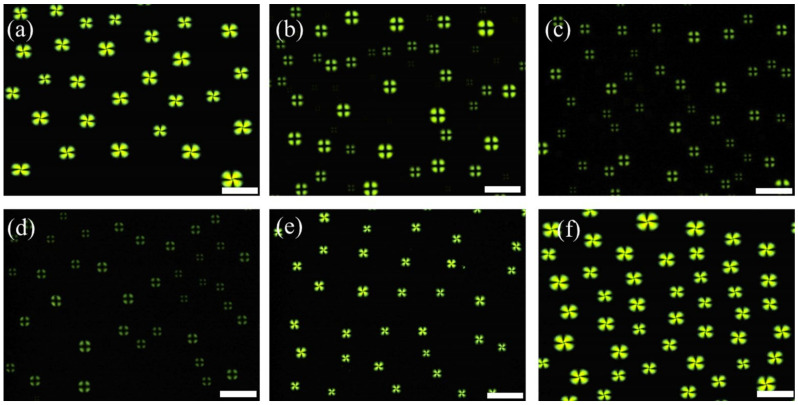
Optical images of liquid crystal (LC) droplets in contact with (**a**) PBS solution, and (**b**) 50 µM, (**c**) 25 µM, (**d**) 10 µM, (**e**) 7.5 µM, and (**f**) 5 µM myristoylcholine chloride (Myr). Scale bar: 100 μm.

In our system, the LC droplet patterns were prepared by spreading 1.5 µL of an anhydrous heptane solution containing 2% (*v*/*v*) 5CB onto the OTS-modified glass slides. Here, Myr was used as a cationic surfactant to induce homeotropic anchoring of LCs at the LC/aqueous interfaces, corresponding to the dark cross appearances of the LC droplets observed under POM. Previous studies have demonstrated that the LC droplet patterns exhibited bright fan-shaped appearances when in contact with non-surfactant solutions (e.g., PBS solution), whereas dark cross textures were observed after introducing surfactant solutions [11,12]. Therefore, we first investigated the orientational behavior of 5CB at the aqueous/LC interface by comparing the optical responses of the LC droplets in contact with PBS and Myr solution. When the LC droplet pattern was immersed in PBS solution (pH = 7.4), we observed bright fan-shaped images coupled to the planar orientation of LCs at the interface (Figure 2a). However, the LCs adopted dark cross textures after the addition of 50 µM Myr solution (Figure 2b), corresponding to the homeotropic orientation of LCs at the aqueous/LC interface. This indicated that the addition of Myr altered the ordering of LCs at the interface from a planar to homeotropic orientation.

Next, we determined the minimum Myr concentration (C_Myr_) to induce homeotropic orientation of the LCs by examining the optical response of LCs after reducing C_Myr_. As shown in Figure 2, dark cross appearances were observed in LCs when C_Myr_ ≥ 10 µM (Figure 2b–d), and bright fan-shaped images were observed when C_Myr_ < 10 µM (Figure 2e,f). Therefore, we determined that the critical C_Myr_ for inducing homeotropic orientation of LCs at the interface was approximately 10 µM.

### 3.2. Feasibility and Detection Limit of the LC Droplet-Based Biosensor for CES Detection

**Figure 3:**
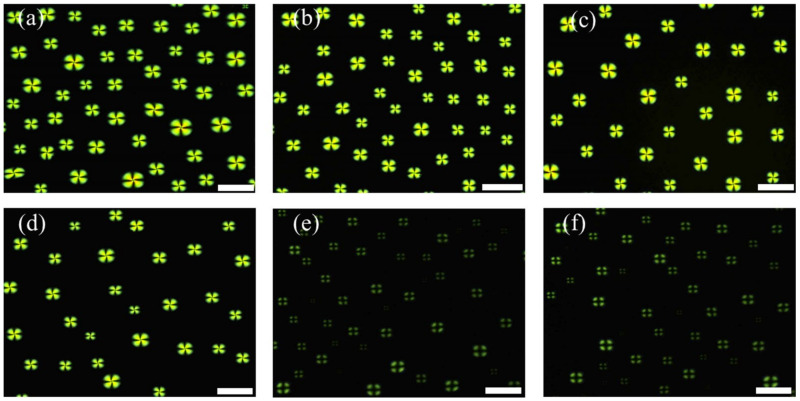
Optical images of liquid crystal (LC) droplets in contact with a pre-incubated mixture of 10 μM myristoylcholine chloride (Myr) and varying concentrations of carboxylesterase: (**a**) 100 mg/L, (**b**) 10 mg/L, (**c**) 1 mg/L, (**d**) 0.1 mg/L, (**e**) 0.01 mg/L, and (**f**) 0.005 mg/L. Scale bar: 100 μm.

According to previous reports, CES can catalyze the hydrolysis of carboxylic acid esters into their corresponding acids and alcohols [21,24]. Thus, to verify the feasibility of the developed sensor, we studied the orientational transition of LCs at the interface after introducing a mixture of Myr and CES. As shown in Figure 3a, bright fan-shaped images were observed after adding the mixture of Myr (10 µM) and 100 mg/L CES to the LC droplet patterns, suggesting a planar orientation of LCs at the interface. This indicated that Myr was completely hydrolyzed by CES and demonstrated that CES activity could be monitored using the developed LC droplet biosensor.

Next, the limit of detection (LOD) for CES was determined by lowering the concentration of CES. The LC patterns showed bright fan-shaped images after incubating with pre-incubated mixtures containing 10 µM Myr and 10 mg/L, 1 mg/L, or 0.1 mg/L CES (Figure 3b–d). However, dark cross appearances were observed when the CES concentration was reduced to 0.01 mg/L or lower (Figure 3e,f), which was attributed to the incomplete hydrolysis of Myr by AChE. Therefore, we concluded that the LOD for CES using this LC droplet sensing system was 0.1 mg/L (2.8 U/L).

### 3.3. Specificity of the LC Droplet-Based Biosensor in CES Detection

**Figure 4:**
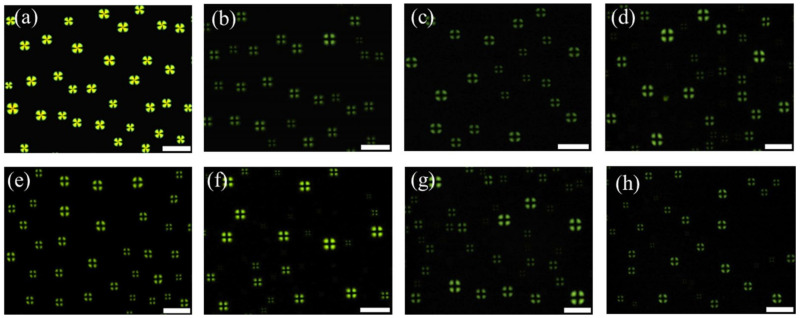
Optical images of liquid crystal (LC) droplets in contact with a pre-incubated mixture of 10 μM myristoylcholine chloride (Myr) and (**a**) 1 μg/mL carboxylesterase (CES), (**b**) 100 μg/mL urease, (**c**) 10 μg/mL lipase, (**d**) 100 μg/mL α-chymotrypsin, (**e**) 1 μg/mL lysozyme, (**f**) 100 μg/mL urea, (**g**) 100 μg/mL glucose, and (**h**) 100 μg/mL CaCl_2_. Scale bar: 100 μm.

The specificity of the developed sensor was also investigated. Various interfering substances, including urease, lipase, α-chymotrypsin, lysozyme, urea, glucose, and CaCl_2,_ were used to test the specificity of the sensor. As shown in Figure 4, a bright fan-shaped texture was observed in the case of CES (Figure 4a), whereas dark cross appearances were obtained for other interfering substances (Figure 4b–h). These results demonstrate the high specificity of the proposed biosensor for CES detection.

**Figure 5:**
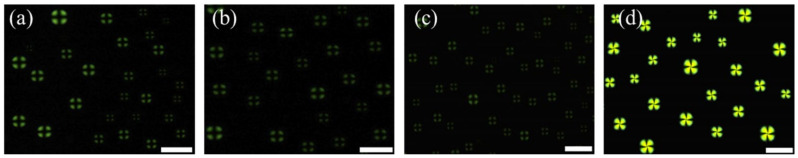
Optical images of liquid crystal (LC) droplets in contact with a mixture of 10 μM myristoylcholine chloride (Myr) and 0.1 mg/L carboxylesterase (CES) pre-incubated with different concentrations of benzil: (**a**) 1 mM, (**b**) 100 μM, (**c**) 10 μM, and (**d**) 1 μM. Scale bar: 100 μm.

It has been reported that benzil is a very potent CES inhibitor and exhibits selective inhibitory effects toward CES [25,26,27]. Therefore, to further confirm the specificity of the CES sensor, we investigated the optical images of LCs coupled with enzymatic hydrolysis of Myr with CES, which was pre-incubated with various benzil concentrations (C_benzil_). Optical images of the LC droplets are presented in Figure 5. Dark cross appearances were obtained when C_benzil_ ≥ 10 µM (Figure 5a–c), indicating that CES activity was prevented by benzil. However, a bright fan-shaped texture was observed when the concentration of benzil was reduced to 1 µM (Figure 5d), suggesting that the concentration of benzil was too low to completely inhibit the activity of CES. These results further confirmed the high specificity of the LC droplet-based biosensor for CES detection. In addition, they also demonstrate the potential application of the LC droplet-based sensing system for the detection or screening of CES inhibitors.

### 3.4. Detection of CES in Human Urine Samples

**Figure 6:**
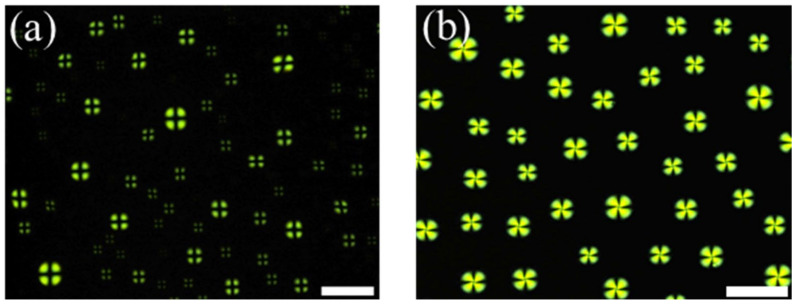
Optical images of liquid crystal (LC) droplets in contact with a mixture of 10 μM myristoylcholine chloride (Myr) and (**a**) human urine (control), (**b**) human urine spiked with 5 mg/L carboxylesterase (CES). Scale bar: 100 μm.

To evaluate the practicability of the developed biosensor, its ability to detect CES in human urine was examined. The human urine was collected from a healthy volunteer. The filtered human urine sample was diluted 10 times with PBS and then spiked with standard CES solution at a concentration of 5 mg/L. The spiked urine samples were analyzed using the developed biosensor. Human urine without the addition of CES was used as a control. The LC pattern displayed a dark cross in the control sample (Figure 6a), confirming there was no CES in the human urine. However, in the urine sample spiked with 5 mg/L CES, bright fan-shaped images were observed (Figure 6b), suggesting that the CES changed the orientation of LCs at the interface. These results prove the potential application of the developed sensing system for CES monitoring in real samples.

Before concluding, we compared the analytical characteristics of the developed LC droplet-based biosensor with other reported methods for CES detection such as spectroscopic, fluorescence, and bioluminescence. These detection methods are limited by several drawbacks such as the need for complex instrumentation, labeling of samples, and long operating times. In contrast, the proposed strategy offers a simple, rapid, label-free, and cost-effective method for the detection of CES with high selectivity. Using this method, a low detection limit of 0.1 mg/L (2.8 U/L) for CES was obtained, which is comparable to or lower than the LODs of other reported methods (Table 1).

## 4. Conclusions

In summary, an LC droplet-based sensing platform was developed to monitor the enzymatic activity of CES, based on the optical responses of LC droplet patterns when in contact with surfactant or non-surfactant solutions. A dark cross appearance was obtained after the introduction of a cationic surfactant solution, Myr, onto the LC droplet patterns, whereas a bright fan-shaped optical image was observed after the addition of a pre-incubated mixture of Myr and CES, owing to the enzymatic hydrolysis of Myr by CES. However, the presence of CES inhibitors, such as benzil, inhibited CES activity resulting in a dark cross appearance. The proposed LC droplet-based biosensor exhibited high specificity toward CES with a low detection limit of 0.1 mg/L (2.8 U/L). In addition, the detection limit of the CES inhibitor was as low as 10 μM. Moreover, the developed biosensor was successfully applied to detect CES in a real human urine sample. This LC droplet-based biosensor not only enables simple, rapid, and label-free detection of CES but also shows high potential for application in the detection or screening of CES inhibitors.

## Figures and Tables

**Table 1:** Comparison of the developed LC droplet-based strategy with other reported methods for carboxylesterase detection.

Methods	Linear Range	Detection Limit	Reference
Spectroscopic probe sensor	40–300 U/L	0.086 U/L	[14]
Near-infrared fluorescence probe	10–300 U/L	3.4 U/L	[15]
Bioluminescence sensor	0.01–6 mg/L	0.01 mg/L	[20]
Surfactant-doped LC-based sensor ^a^	18–180 U/L	1 mg/L (18 U/L)	[21]
LC droplet-based biosensor	-	0.1 mg/L (2.8 U/L)	This study

^a^ LC, liquid crystal.

## Supplementary Materials

Supporting information can be downloaded at: https://www.mdpi.com/article/10.3390/mi13030490/s1.
