# Supplementary material for: Simple and Label-Free Detection of Carboxylesterase and Its Inhibitors Using a Liquid Crystal Droplet Sensing Platform
Source: Micromachines (Basel). 2022 Mar 21;13(3):490. doi: 10.3390/mi13030490 (PMC8954150; doi:10.3390/mi13030490)
Supplement: Supplementary file 1 [file micromachines-13-00490-s001.zip › micromachines-1645178-supplementary.pdf]

Supplementary Material

Simple and sensitive detection of carboxylesterase and its inhibitors using a liquid crystal droplet sensing platform

Duy-Khiem Nguyen and Chang-Hyun Jang *

Department of Chemistry, Gachon University, Seongnam-daero 1342, Sujeong-gu, Seongnam-si, Gyeonggi-do 13120, Republic of Korea.

*Corresponding author: Tel. +82–31–750–8555; fax: +82 31 750 8774

E-mail address: chjang4u@gachon.ac.kr (C.-H. Jang)

Materials and Apparatus

Nematic liquid crystals of 4-cyano-4′-pentylbiphenyl (5CB) and benzil were obtained from Tokyo Chemical Industry Co., Ltd. (Tokyo, Japan). Glass slides were obtained from Matsunami Glass IND., Ltd. (Osaka, Japan). Myristoylcholine chloride (Myr) was provided by Toronto Research Chemicals Inc. (Toronto, Canada). Carboxylesterase (from porcine liver, 28 units/mg), octyltrichlorosilane (OTS), phosphate-buffer saline (PBS, pH 7.4), urease (from *Canavalia ensiformis*, 50 units/mg), lipase (from porcine pancreas, 500 units/mg), α-chymotrypsin (from bovine pancreas, 57.24 units/mg), lysozyme (from chicken egg white, 100,000 units/mg), urea, glucose, and $CaCl_2$ were purchased from Sigma-Aldrich (St. Louis, MO, USA). n-Heptane (anhydrous), H_2O_2 (30% w/v), H_2SO_4 (95%), C_2H_5OH, CH_3OH, and CH_2Cl_2 were purchased from Daejung Chemicals & Metals Co., Ltd. (Daejung, South Korea). Deionized (DI) water (18.2 MΩ.cm) was generated using a Milli-Q water purification system (Millipore, Bedford, USA).
A polarized light microscope (POM, Eclipse LV100 POL; Nikon, Japan) was used to capture optical images of the liquid crystals (LCs). All images were captured using a digital camera (DS–2Mv; Japan) mounted on a POM with 10× objective lenses in the transmission mode at a resolution of 2560 × 1920 pixels.

Preparation of OTS-modified glass slides

Briefly, microscope glass slides were immersed in "piranha solution" (H_2SO_4/H_2O_2 = 7/3 [v/v]) at 80°C for 2 h. The slides were rinsed with deionized (DI) water, ethanol, and methanol and dried under a nitrogen stream, and stored overnight at 120°C. The piranha-treated glass slides were then immersed in an OTS/n-heptane (150 μL/135 mL) solution at 25 °C for 1 h. The glass slides were then washed with dichloromethane and dried under nitrogen flow.
